# Innate Immune Cells: Monocytes, Monocyte-Derived Macrophages and Microglia as Therapeutic Targets for Alzheimer’s Disease and Multiple Sclerosis

**DOI:** 10.3389/fncel.2019.00355

**Published:** 2019-07-31

**Authors:** Adham Fani Maleki, Serge Rivest

**Affiliations:** Neuroscience Laboratory, CHU de Québec Research Center and Department of Molecular Medicine, Faculty of Medicine, Laval University, Québec City, QC, Canada

**Keywords:** microglia, monocytes, macrophages, brain diseases, innate immune response, phagocytosis, neuroinflammation

## Abstract

The immune system provides protection in the CNS via resident microglial cells and those that traffic into it in the course of pathological challenges. These populations of cells are key players in modulating immune functions that are involved in disease outcomes. In this review, we briefly summarize and highlight the current state of knowledge of the differential contributions of microglia and monocytes in Alzheimer’s disease and multiple sclerosis. The role of innate immunity is frequently seen as a Yin and Yang in both diseases, but this depends on the environment, pre-clinical disease models and the type of cells involved.

## Introduction

Innate immunity is not limited to the first line of defense during infections, but also plays a crucial role in the clearance of cellular debris and tissue repair. The central nervous system (CNS) contains various types of myeloid cells, namely mast cells (Hendriksen et al., 2017; Lenz and Nelson, 2018), parenchymal microglia, perivascular macrophages, meningeal macrophages, choroid plexus macrophages and blood-borne monocytes (Thériault et al., 2015). Regardless of the polarization state, microglia activation directs the inflammatory response in almost all neurodegenerative diseases (Colonna and Butovsky, 2017). Upon immune challenge or brain diseases, microglia undergo extreme morphological changes, which are associated with the release of cytokines, chemokines and/or trophic factors (Reviewed in details in ElAli and Rivest, 2016).

On the other hand, perivascular, meningeal and choroid plexus macrophages may modulate immune cell entry and phenotype during inflammation. More precisely, several reports point out their implication in various immunopathological processes, including antigen presentation to circulating lymphocytes (Haass and Selkoe, 2007; Jack et al., 2013; Michaud et al., 2013a). Perivascular macrophages sense blood danger signals, including damage-associated molecular patterns (DAMPs), and pathogen-associated molecular patterns (PAMPs) under steady-state conditions (Goldmann et al., 2016). On the same conditions, choroid plexus macrophages are believed to surveil CSF production (Michaud et al., 2012).

Several recent reviews have comprehensively discussed the roles of the immune system in the CNS. Here, we provide an overview of some recent progresses as well as emphasizing the high therapeutic potential of microglia, monocytes and monocyte-derived macrophages in CNS disorders, such as Alzheimer’s disease (AD) and multiple sclerosis (MS).

## Microglia and Monocyte Ontology

Microglia play essential roles in monitoring and performing rapid response to changes in CNS and constitute the main resident immune cell population of the brain (Soulet and Rivest, 2008). Although their origin has remained controversial for a long time, recent studies have reported that microglia are derived from embryonic yolk sac progenitors (Alliot et al., 1999; Ginhoux et al., 2010; Kierdorf et al., 2013; Hagemeyer et al., 2016). Maintenance of microglia in adult CNS is a matter of debate, but to date it is an accepted notion that in physiological conditions microglia are a self-renewal cell population and postnatal hematopoietic progenitors are not involved (Goldmann et al., 2016). Regardless of their exact origin and their maintenance, it is widely accepted that these cells are never resting and perform a continuous surveillance in brain (Kettenmann et al., 2013). Depending on the nature of treat, microglia activation will release a large family of molecular mediators that can either be protective via phagocytosis or be detrimental via their neurotoxic effects (Kettenmann et al., 2013; Lampron et al., 2013).

On the other hand, monocytes arise from self-renewal hematopoietic stem cells and progenitor cells located in the bone marrow (BM) (Geissmann et al., 2010). CNS contains tissue macrophages such as perivascular, meningeal and choroid plexus macrophages. Recent study demonstrated that these macrophages originate from early yolk-sac, similar to microglia, and that their maintenance does not depend on circulating monocytes except the choroid plexus macrophages that may be replenished by blood cells (Goldmann et al., 2016). Although all CNS macrophages share common origin (yolk sac and/or BM) (Schulz et al., 2012; Xu et al., 2015; Goldmann et al., 2016) and share numerous common myeloid- and macrophage-specific markers, they exhibit quite diverse and cell-specific properties and functions (Michaud et al., 2013a; Shemer et al., 2015).

## Immune Regulation and Function of Microglia and Mononuclear Phagocytic Cells in AD

Alzheimer’s disease is the most common cause of dementia worldwide, a neurodegenerative disease that is accompanied with a chronic activation of innate immune cells within the CNS. The pathogenesis of AD is associated with the accumulation of amyloid beta (Aβ) in the parenchyma and cerebral vasculature due to impaired clearance of the neurotoxic Aβ_*1–40*_ and Aβ_*1–42*_ peptides. The cerebral accumulation of Aβ peptides, causes a sustain and chronic inflammatory response, tau hyperphosphorylation, synaptotoxicity, and therefore neuronal loss, accelerating the progressive cognitive decline (Haass and Selkoe, 2007; Jack et al., 2013). Epidemiological and experimental studies show the crucial role of the innate immune system in AD. More precisely, several studies demonstrated a differential roles of microglia, monocytes and cerebral macrophages in Aβ pathology outcome (Michaud et al., 2012; Prokop et al., 2013). The significant of microglia and macrophages in systemic inflammation and neurodegenerative diseases have been discussed comprehensively by Perry et al. (2007). Briefly, they discussed the concept of primed microglia which is a type of microglia that responds to pathological changes in the CNS and consequently becomes more susceptible to activation. They proposed that systemic inflammation affects these primed microglia, possibly contributing to disease progression in chronic neurodegenerative diseases (Perry et al., 2007).

Previous studies have shown that a deficit in phagocytosis together with secretion of inflammatory cytokines are harmful to neurons and favor Aβ accumulation in both the brain parenchyma and the neurovascular unit (Prokop et al., 2013; Orre et al., 2014). Therefore, for a long time, aberrant chronic inflammation was considered to act as a pivotal element to promote AD. Nevertheless, several clinical trials failed to show beneficial effects of anti-inflammatory agents in AD patients, even worsen the outcome in some cases (Prokop et al., 2013). Recent studies have shown that inhibition of key inflammatory mediators were found to improve pathology (Heppner et al., 2015; Daniels et al., 2016). In addition, overexpression of the microglial triggering receptor expressed on myeloid cells 2 (TREM2) improves the cognitive decline in mouse models of AD (Jiang et al., 2016) and TREM2 deletion aggravates pathology (Xiang et al., 2016; Mecca et al., 2018). Moreover, a rare mutation of human TREM2 (R47H) determines a high risk for developing AD and Nasu-Hakola disease, a disorder characterized by dementia, and amyloid plaque deposition is caused by genetic mutation of TREM2 or DAP12 genes (Paloneva et al., 2002). TREM2 is a microglial surface receptor that interacts with DAP12. Involvement of TREM2-DAP12 pathway in AD (and other neurodegenerative diseases) has been extensively reviewed and discussed by Mecca (Mecca et al., 2018). Briefly, TREM2 or DAP12 deficiency may lead to deficient microglial activation without effective amyloid clearance and consequently neurodegeneration and plaque deposition (Mecca et al., 2018). These results may highlight the need for a finely tuned innate immune activation, perhaps achieved by a moderate and time-dependent activation of microglia and/or monocytes. In this regard, we have demonstrated that stimulation of phagocytosis without a constant excessive inflammatory response has robust beneficial effects. More precisely, peripheral administration of a detoxified TLR4 ligand called monophosphoryl lipid A which is a lipopolysaccharide derivative, improved cognitive deficits in mouse model of AD (Michaud et al., 2013b). Similarly, macrophage colony-stimulating factor (mCSF) treatment as a molecule that modulates monocyte and microglial proliferation and activity reduced Aβ levels and improved cognitive function in the brain parenchyma (Boissonneault et al., 2009). These observations may also highlight that time is everything – early activation of microglia and monocytes is highly beneficial while late treatment with these molecules is not as effective and may also further accelerate AD progression. Taken together, these studies pointed out a great therapeutic potential of molecules that activate innate immune cells in a very specific direction, which is a marked phagocytic capacity with a low inflammatory reaction.

**FIGURE 1:**
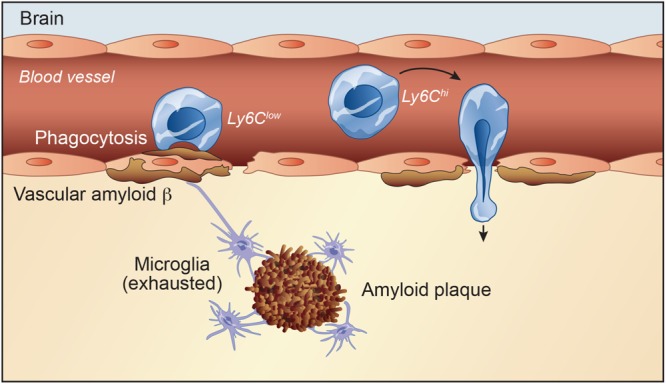
Initially, defects in Aβ clearance pathways lead to accumulation of the peptide in the brain leading to neuronal toxicity. Activated microglial cells are capable of performing several macrophage-like immune functions, such as cytokine release and phagocytosis of Aβ. However, exhausted microglia are a common signature in AD. Majority of AD patients suffer from cerebrovascular dysfunction, which compromises the integrity of the blood-brain barrier (BBB). A compromised BBB promotes the entry of infiltrating cells within the perivascular space and brain parenchyma and infiltrating cells may exacerbate or alleviate disease progression. Mononuclear phagocytic cells can enter and become involved in CNS pathological situations. Under pathophysiological conditions, Ly6C^*high*^ monocytes adhere to brain endothelium and consequently infiltrate brain parenchyma. Ly6C^*low*^ monocyte subset associates with Aβ-positive veins, but not arteries, internalizes Aβ, and efficiently eliminates Aβ microaggregates and transports them from the brain microvasculature to the blood circulation.

The CNS contains an extensive vasculature network with circulating monocytes, granulocytes and dendritic cells. During certain diseases involving BBB leakage, infiltrating cells may exacerbate or alleviate disease progression (Figure 1). In parallel, dysfunctional microglia are a common signature in many pathological conditions (Prinz et al., 2011; Prinz and Priller, 2014). Thus, using monocytes and macrophages to compensate what endogenous microglial cells can no longer achieve efficiently is therapeutically promising (Prinz et al., 2011). Majority of patients with AD suffer from cerebral amyloid angiopathy (CAA) as result of Aβ deposition in leptomeningeal and cortical blood vessels. Accumulating evidence suggests that vascular amyloid deposits may result from impaired clearance of neuronal Aβ along perivascular spaces (Thériault et al., 2015). In this regard, Hawkes and McLaurin (2009) reported that depletion of perivascular macrophages significantly increased the vascular Aβ levels. Conversely, stimulation of perivascular macrophage turnover reduced cerebral CAA load, highlighting the importance of perivascular macrophages in this brain disease (Hawkes and McLaurin, 2009). In parallel, a recent study highlighted a key role of the choroid plexus in AD pathology and how it may affect the blood-cerebrospinal fluid barrier, and immune function. They identified a high-risk haplotype: ApoE ε4, Tau/H1, and TREM2/T. In addition, in AD and Down syndrome brains, they showed insoluble tau and ApoE accumulation in the choroid plexus that may increase rate of Aβ_*42*_ oligomerization and impair tau trafficking (Raha-Chowdhury et al., 2019).

Microglia have been known as modulator of neuroinflammation and release of neurotoxic molecules. Nonetheless, recent studies have extended our perception about microglia by providing evidences supporting direct role of microglia-mediated synapse loss independently of Aβ in AD. This phenomenon is discussed comprehensively by Rajendran and Paolicelli (2018) considering both genetics and life style factors. Briefly, the discussed previous observations suggest that the different ApoE isoforms critically affect the levels of synapse loss and is associated with increased microgliosis and astrocytosis, as well as relationship between sleep quality and risk for AD (Rajendran and Paolicelli, 2018).

Several studies reported that mononuclear phagocyte cells enter and become involved in CNS pathological situations through microglial secretion of various cytokines and chemokines in a CCR2 signaling manner (Mildner et al., 2009; Zhang et al., 2013). In human, monocytes are regrouped into three main subsets based on their CD14 and CD16 expression levels, which are the classical subset (CD14^++^CD16^–^), the intermediate subset (CD14^++^CD16^+^) and the non-classical subset (CD14^+^ CD16^++^) (Auffray et al., 2009). In mice, monocytes are regrouped into two main subsets based on chemokine receptors and Ly6C expression levels; namely the pro-inflammatory subset (CX3CR1^*low*^CCR2^+^Ly6C^*high*^) which is involved in inflammatory responses, and the anti-inflammatory subset (CX3CR1^*high*^CCR2^–^Ly6C^*low*^) that establishes the resident patrolling monocyte population (Naert and Rivest, 2013). Ly6C^*low*^ monocytes is considered as the resident phagocyte population that patrols the lumen of blood vessels and enhances tissue repair (Naert and Rivest, 2013). Pro-inflammatory monocytes have been shown to infiltrate the brain and differentiate into activated macrophages. These BM-derived macrophages are involved in phagocytosis of toxic elements, including Aβ, and are known to be more efficient than resident microglia in clearing cerebral Aβ deposits in AD models (Thériault et al., 2015). Generally, monocytes are considered more efficacious than resident microglia in Aβ clearance. Several reports outlined the importance of these anti-inflammatory monocytes in AD. Indeed, recently a study reported a reduction in the non-classical CD14^+^CD16^++^ monocytes in AD patients compared with mild cognitive impairment patients or age-matched healthy controls (Saresella et al., 2014). In addition, using the two-photon microscopy, our group demonstrated that the patrolling monocyte subset associated with Aβ-positive veins, but not arteries, internalize Aβ, and efficiently eliminate Aβ microaggregates and transport them from the brain microvasculature to the blood circulation (Figure 1; Michaud et al., 2013a).

## Immune Regulation and Function of Microglia and Mononuclear Phagocyte Cells in MS

Multiple sclerosis (MS) is an autoimmune inflammatory disorder of the CNS leading to damage the integrity of the axons and myelin sheaths as well as neurological disabilities. Peripheral immune cells play crucial role in onset of the disease. In addition to lymphocytes, other myeloid cells infiltrate the CNS and initiate neuroinflammation. These cells comprise parenchymal microglia, circulating monocytes and monocyte-derived macrophages (Dendrou et al., 2015).

**FIGURE 2:**
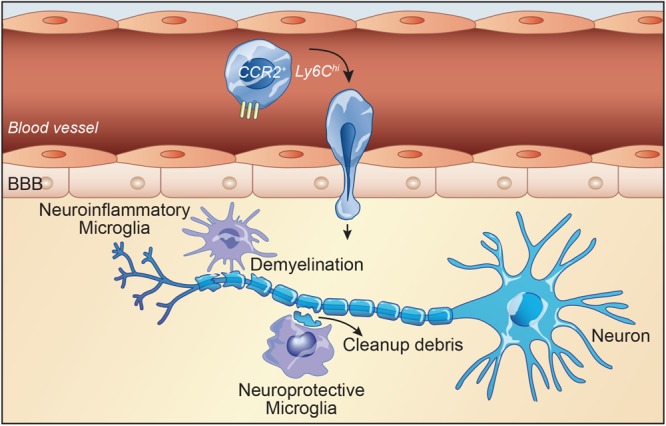
Inflammatory monocytes play central role in disease progression in experimental autoimmune encephalomyelitis (EAE). In the early stages of EAE, myeloid cells, mainly monocytes, infiltrate the CNS and contribute to the inflammatory response and pathology. Chemokine receptor CCR2 is critical for the accumulation of Ly6C^*high*^/CCR2^*high*^ monocytes in the CNS. CCR2 and its ligand CCL2 play an important role in regulating Ly6C^*high*^/CCR2^+^ monocyte infiltration to the CNS and facilitate tissue damage in multiple sclerosis (MS) and EAE. On the contrary, inhibition of monocyte recruitment delays the disease. Microglia and macrophages accumulate in active sites of demyelination and neurodegeneration in MS. The majority of cells associated with active demyelination originate from resident microglia. Reactive microglia are present at early and late stages of disease. They are present in demyelinating lesions. Microglial activation even occurs before the onset of EAE. Once microglia are activated, they can damage other CNS cells, in particular oligodendrocytes and neurons. Microglia activation induces massive immune cell infiltration and demyelination which is followed by remyelination. However, microglia have been shown to exhibit both neuroinflammatory as well as neuroprotective effects.

In MS, proinflammatory cytokines such as tumor necrosis factor (TNF) α, IL-6, and IL-1β activate microglia (Jiang et al., 2014). Microglial activation in turn increase various proinflammatory factors which consequently lead to exacerbation of disease symptoms (Rawji and Yong, 2013). Generally, microglia perform beneficial roles specially during remission phase. Yamasaki et al., have shown that microglia have the ability to clean up myelin debris and also enhancing tissue repair (Auffray et al., 2009). In addition, Kocur et al. (2015) have reported microglia activation and IFNγ production along with improvement of pathology by clearance of myelin debris. In parallel, aged animals failed to remyelinate together with a reduced clearance of myelin debris (Ruckh et al., 2012). Our group has shown that inefficient clearance of myelin debris in mice with CX3CR1 deficiency impairs the integrity of myelin sheaths and thus preventing remyelinating processes (Lampron et al., 2015). Recent study from our group demonstrated that mCSF-treated cuprizone-fed mice reduced myelin loss during the demyelination phase with an increased number of microglia in lesion sites. In addition, tamoxifen-induced conditional deletion of the mCSF receptor in microglia from cuprizone-fed mice caused aberrant myelin debris accumulation and reduced microglial phagocytic response (Laflamme et al., 2018). In MS and experimental autoimmune encephalomyelitis (EAE) model, microglial activation induces massive immune cell infiltration and demyelination which is followed by remyelination. Overall, microglia have been shown to exhibit both neuroinflammatory as well as neuroprotective effects (Figure 2). However, infiltrating monocytes are generally known to have more detrimental effects in the disease course and severity (Mishra and Yong, 2016).

In EAE, high expression of CD40, CD80, CD86, inducible nitric oxide synthase (iNOS), Ly6C, and MHCII in monocyte-derived macrophages demonstrate they are mostly proinflammatory cells (Jiang et al., 2014). More importantly, these proinflammatory monocyte-derived macrophages accumulate in the onset and peak disease phases (Jiang et al., 2014; Moline-Velazquez et al., 2016). Consequently, decrease in macrophage densities and shifting macrophage population toward an anti-inflammatory phenotype was correlated with improvement in clinical remission phase (Jiang et al., 2014).

Due to the crucial role of monocytes in MS pathology, there is growing interest to target them for a potential MS treatment. CCR2 and its ligand CCL2 regulate infiltration of monocytes in the CNS (Hulkower et al., 1993). Indeed, lack of CCR2, or deletion or inhibition of CCR2 reduced monocyte-derived macrophage recruitment in the CNS of EAE mice that consequently exhibit less severe disease scores (Figure 2; Dogan et al., 2008; Hsieh et al., 2014; Moreno et al., 2014). On the contrary, enhancing infiltration of proinflammatory monocytes via SOCS3 deficiency in EAE accelerates onset and worsens disease state (Qin et al., 2012). This result clearly shows that inflammatory monocyte recruitment relies heavily on CCR2-mediated chemotaxis. Moreover, microglia-specific ablation of transforming growth factor (TGF)-beta-activated kinase-1 in CX3CR1^+^ macrophages significantly suppresses EAE and decreased axonal damage. More precisely, EAE is suppressed by a cell-dependent inhibition of NFkB, JNK, and ERK1/2 pathway, indicating a clear contribution of monocytes and differentiated macrophages to pathology progression (Goldmann et al., 2013). A recent study provided evidence that CX3CR1 might be an important regulator of MHC-II expression in antigen-presenting cells, playing a beneficial role in EAE. They showed beneficial role of CX3CR1 in a chronic EAE model via modulation of infiltrating myeloid cells (Mai et al., 2019).

The mechanism of monocyte recruitment remains relatively elusive. Some studies showed that leukocyte infiltration into CNS requires matrix metalloproteinases (MMPs), as evidences obtained from EAE and *in vitro* showed that monocytes, macrophages and microglia express a variety of MMPs (Yong et al., 2001; Nuttall et al., 2007). In addition, monocytes, infiltrated macrophages and microglia in EAE exhibit elevated expression levels of CD147 which is an upstream regulator of MMP expression (Agrawal et al., 2011). Interestingly, significant of MMPs and CD147 expression levels in disease progression have been demonstrated by inhibition of CD147 activity (Agrawal et al., 2011).

As discussed above, Microglia and infiltrating myeloid cells play dual roles in the disease course. Indeed, microglial activation is related to disease development (Rawji and Yong, 2013). Nevertheless, Microglia is involved in remyelination due to its phagocytic ability, secretion of neuroprotective, anti- inflammatory molecules and P2X4R signaling (Zabala et al., 2018). Dual roles of microglia and other phagocytic cells are also represented in other studies indicating that proinflammatory and regulatory markers expressed in these cells throughout the course of an active demyelinating MS lesions (Peferoen et al., 2015; Zabala et al., 2018). Moreover, active demyelination mediated by infiltrating monocytes is also associated with reactive microgliosis (Lassmann, 2014). Therefore, it seems crucial to develop specific markers to distinguish between myeloid cells from morphological and functional point of views to identify their complex roles in the inflammatory/degenerative cascade.

Several research groups made effort for developing markers, such as P2RY12 and TMEM119 (Butovsky et al., 2014; Bennett et al., 2016; Satoh et al., 2016). Other recent studies were looking for new markers that are stable in different pathological stage of EAE. In this regard, Ajami et al. (2018) performed high-dimensional analysis of cell surface markers, signaling molecules and cytokines on brain myeloid cells at the single-cell level using mass cytometry in EAE, and mouse models of Huntington’s disease (HD) and amyotrophic lateral sclerosis (ALS) (Ajami et al., 2018). They found three populations of myeloid cells exclusive to the CNS, which were present in each disease model. In contrast, blood-derived monocytes comprised five populations and migrated to the brain in EAE (but not in HD and ALS models). Their results confirmed expression of recently identified microglial markers 4D4 and FCRLS in resident microglia (Butovsky et al., 2014). In addition, they found in EAE (but not the two other models) that two of the CNS-resident myeloid populations developed a closely coordinated series of signaling events with pCREB and pMAPKAPK2 as their signature (Ajami et al., 2018). These results may illustrate very interesting differences between cell subsets in brain diseases mediated by strong and classical inflammatory response (e.g., EAE) Vs those that are not (e.g., HD and ALS). Moreover, they observed that in EAE, peripheral myeloid cells and resident myeloid cells have similar cytokine expression profiles. TNFα being the most prominently cytokine produced in the three CNS myeloid cell populations followed by IL-6, GM-CSF, IL-10, and TGF-β (Ajami et al., 2018).

In parallel to this study, another interesting new report identified different subsets of myeloid cells and phenotypic changes in EAE (and also in AD) (Mrdjen et al., 2018). Using Sall1^*GFP*^ reporter mice, they distinguished between resident microglia form other myeloid cells. They showed a decrease of CD14 together with higher MHCII and Sca-1 expression levels in microglia of EAE mice in comparison to the groups of aged and AD mice. Additionally, their results demonstrated that phenotypic change in microglial population in EAE model is homogenously, while this was observed just in small subset of microglia in aging and AD models. They also observed that monocyte-derived cells (differentiated from Ly6C^*high*^ monocytes) display a homogenous macrophage-like phenotype. In addition, they introduced CD38 and MHCII as markers for distinguishing CNS macrophage subsets (Mrdjen et al., 2018).

Macrophage accumulation in MS lesion is positively correlated with active demyelination. In reducing macrophage infiltration and activation, minocycline administration attenuated clinical scores in EAE (Niimi et al., 2013). Another study in EAE showed that inhibition of monocyte recruitment into the lesion site was able to delay disease onset and slightly reduce severity of disease (Saederup et al., 2010). The significance of monocyte-derived macrophages is not limited to disease initiation. Indeed, more recent studies extended our understanding by pointing out their implication in remyelination and demyelination. Miron et al. (2013) reported that pro-inflammatory macrophages were required for progenitor proliferation, but dispensable for efficient remyelination. Conversely, specific depletion of the M2 macrophage population using mannosylated clodronate liposomes significantly impaired progenitor differentiation into myelinating oligodendrocytes and remyelination (Miron et al., 2013).

Macrophages in the choroid plexus, perivascular space, and the meninges, (as well as monocytes from the blood) that crossing the damaged BBB become monocyte-derived macrophages (Lucchinetti et al., 2000) may show subtype-specific effector functions in the diseased CNS. Recent study described macrophages in CNS interfaces from origin, fate and dynamic point of views. Briefly, they provided evidences indicating that generation of CNS microphages relied on the transcription factor PU.1 but not BATF3 and NR4A1 transcription factors. They showed that while non-parenchymal CNS macrophages are so similar to microglia, they are still distinguished population of tissue macrophages with specialized functions. In agreement with other studies, their results highlighted fundamental involvement of the choroid plexus in the development and progression of MS and its animal model EAE (Goldmann et al., 2016). Another recent study from this team is a comprehensive characterization of myeloid subsets in several CNS compartments during neuroinflammation. Combining fate mapping, deep single-cell transcriptome analysis, clonal analysis, *in vivo* imaging and transgenic mouse lines, they reported self-renewal, and random proliferation shifts toward clonal expansion in CNS macrophages upon inflammation (Jordão et al., 2019). These result may imply that different types of CNS macrophages indicate the differential macrophage response to inflammation, suggesting functional diversity of these cells during disease (Jordão et al., 2019). Moreover, previous report showed that CNS macrophages have considerable longevity and remain stable even during CNS autoimmunity (Ajami et al., 2011). On the other hand, blood-derived myeloid cells, presumably monocytes, infiltrated the spinal cord during disease but were only transiently present at the lesion site. This study confirmed the previous report utilizing the proper fate-mapping tools (Jordão et al., 2019). Furthermore, important roles of infiltrating myeloid cells in the activation of T cells were found during EAE in mice exhibiting specific MHCII deficiency in CNS macrophages. Indeed, they discovered no changes, either in the clinical course or in the histopathology of EAE mice in this model since T cells preferentially show long lasting interactions with peripheral myeloid cells when they express MHCII (Jordão et al., 2019).

## Concluding Remarks

Innate immunity plays a central role in neuroinflammatory diseases. The role of all populations of innate immune cells in brain disorders is far from being black and white and this depends on the environment, cell populations, time of disease and models. Both resident and systemic myeloid cells can have robust beneficially and detrimental properties in AD and MS. The proper molecules, time of treatment and the environment will 1 day tell us how to manipulate these cells in order to prevent, treat or even cure these supposedly Yin and Yang inflammatory diseases.

## Author Contributions

Both authors wrote the manuscript together.

## Conflict of Interest Statement

The authors declare that the research was conducted in the absence of any commercial or financial relationships that could be construed as a potential conflict of interest.
